# Leaf Developmental Stage at Harvest Affects Postharvest Senescence and Quality Parameters of Kale

**DOI:** 10.3390/metabo16090684

**Published:** 2026-09-17

**Authors:** María Clara Donadelli, Estefanía Bernay, Gricel Alejandra Cagliardi, Gustavo Martínez, Victoria Casajus

**Affiliations:** 1 Laboratorio de Investigación en Productos Agroindustriales, Facultad de Ciencias Agrarias y Forestales, Universidad Nacional de La Plata, Calle 60 y 119, La Plata 1900, Buenos Aires, Argentina; m.claradonadelli@gmail.com; 2 Facultad de Ciencias Agrarias y Forestales, Universidad Nacional de La Plata, Calle 60 y 119, La Plata 1900, Buenos Aires, Argentina; 3 Instituto de Fisiología Vegetal (INFIVE), Universidad Nacional de La Plata—CONICET, Diagonal 113 and 61, La Plata 1900, Buenos Aires, Argentina; bernay.estefania@gmail.com (E.B.); martga@gmail.com (G.M.); 4 Facultad de Ingeniería, Universidad Nacional de La Plata, Calle 1 y 47, La Plata 1900, Buenos Aires, Argentina

**Keywords:** kale, postharvest, leaf developmental stage, chlorophyll degradation, nutritional quality

## Abstract

**Highlights:**

**What are the main findings?**

• Leaf developmental stage at harvest affected kale postharvest senescence and metabolite composition.
• Inner leaves retained a greener color and higher chlorophyll, protein, and reducing sugar contents during storage, while outer leaves showed an earlier activation of chlorophyll catabolism genes.

**What are the implications of the main findings?**

• Leaf developmental stage at harvest is an important determinant of kale postharvest senescence.
• Selecting inner leaves at harvest may contribute to improved quality retention during storage.

**Abstract:**

**Background**: Kale is a vegetable of the *Brassicaceae* family recognized for its high nutritional value. After harvest, kale leaves undergo senescence, characterized by the loss of green color due to chlorophyll degradation. This deterioration affects organoleptic quality and consumer acceptance. **Objective:** The present study investigated the effect of leaf developmental stage at the moment of harvest on postharvest senescence and parameters related to the nutritional quality of kale during postharvest storage at 20 °C. **Material and Methods:** Kale leaves were harvested at three developmental stages—young inner leaves (~20 cm), middle leaves (central position, 20–30 cm), and mature outer leaves (35–40 cm)—and stored at 20 °C for six days in darkness with an RH of 90–95%. At harvest and after two, four, and six days of storage, superficial color, weight loss, total phenolic and flavonoid contents, antioxidant capacity, total and reducing sugars, and total and soluble proteins were evaluated. Because yellowing is the main postharvest symptom in kale leaves, the relative expressions of chlorophyll catabolic genes such as *NYC*, *NOL*, *SGR*, *PPH* and *PaO* were also analyzed. **Results**: The leaf development stage strongly influenced postharvest life and the biochemical composition of the leaves. Inner leaves showed a lower rate of chlorophyll degradation during postharvest storage and retained a more intense green color, consistent with a reduced expression of chlorophyll catabolic genes. In contrast, middle and outer leaves exhibited greater chlorophyll loss and more pronounced yellowing. Inner leaves maintained the best overall visual quality at the end of storage and showed higher phenolic as well as greater antioxidant capacity compared with middle and outer leaves. Inner leaves also contained higher levels of reducing sugars and soluble proteins. **Conclusions**: Taken together, these findings indicate that harvesting kale at earlier developmental stages might represent an effective strategy to extend shelf-life and provide a product with improved values in the parameters actually measured.

## 1. Introduction

Kale is a green leafy vegetable belonging to the *Brassicaceae* family. This vegetable has gained special attention for its high content of nutritional compounds important for a healthy diet, such as phenols, flavonoids, glucosinolates, vitamins, minerals, and dietary fiber [1,2]. The consumption of kale is associated with a lower risk of several diseases such as cancer and inflammation due to its antioxidant, anticancer, cardiovascular, and gastrointestinal system activities [3]. These large amounts of health-promoting phytonutrients make kale an increasingly consumed vegetable with a high-growth trend in global production and cultivation [4]. However, the concentration of these phytochemicals may change rapidly after harvest as a consequence of leaf senescence, which can reduce both nutritional and commercial quality [5].

Kale leaves can be harvested between four and six weeks after sowing, depending on the appropriate size and texture of the leaves. Harvesting is done by hand picking the older outer leaves to allow the plants to regrow and produce more leaves, or by cutting the whole plant. This latter practice involves harvesting leaves with different developmental stages. The marketability of kale depends on its leafy green color, so the chlorophyll degradation that occurs during postharvest storage is the main problem for the commercialization of this vegetable [6]. After harvest, the leaves begin to turn yellow due to chlorophyll degradation and lose weight, reaching a shelf-life of about seven to ten days when they are stored at room temperature [7]. The decrease in the content of chlorophylls is regulated by chlorophyll catabolic genes (CCGs) such as *NYC*, *NOL*, *SGR*, *PPH*, and *PaO*. These are the main genes involved in the chlorophyll degradation pathway in the *Brassicas* genus, converting the chlorophyll molecules into chlorophyll degradation products [8]. Briefly, chlorophyll b is first converted into chlorophyll a by Chl b reductase, encoded by *NYC1* and *NOL* genes. Subsequently, chlorophyll a is converted into pheophytin a by the *SGR* gene. Pheophytin a is then hydrolyzed by pheophytinase (PPH), producing pheophorbide a and phytol. Pheophoribide a is subsequently cleaved by pheophorbide a oxygenase (PaO), generating the oxidized red chlorophyll catabolite, which is further reduced by red chlorophyll catabolite reductase to produce the primary fluorescent chlorophyll catabolite. This is converted into non-fluorescent chlorophyll catabolites under acidic conditions in the vacuole [9] (Supplementary Figure S1).

Leaves located at different positions within the canopy differ in their physiological age, photosynthetic activity, source–sink relationships, and antioxidant metabolism. Consequently, they may exhibit different responses to postharvest stress and senescence. Various preharvest factors, such as agronomic practices, cultivars, climatic conditions, time of harvest and maturity at harvest, are known to influence the shelf-life of green leafy vegetables [10]. Among them, it has been observed that the leaf developmental stage at the moment of harvest can influence the quality of the product during postharvest storage. In the case of rocket leaves, it has been observed that age influenced metabolic activity but had no importance for postharvest quality [11]. In other Brassicas species, differences in quality and in the postharvest rate of senescence have been observed in primary or secondary inflorescences during the postharvest refrigeration of fresh-cut broccoli [12]. In addition, leaf maturity at harvest had an important effect on postharvest senescence at 5 °C in fresh-cut kale leaves [13]. Although previous studies have shown that harvest maturity can influence the postharvest performance of vegetables, little information is available regarding the effect of leaf developmental stage on chlorophyll catabolism, nutritional quality and the molecular regulation of senescence in kale leaves stored at room temperature. Therefore, the aim of this study was to determine how leaf developmental stage at harvest affects postharvest senescence, chlorophyll degradation, some parameters related to nutritional quality, and the expression of chlorophyll catabolic genes in kale leaves stored at 20 °C.

## 2. Materials and Methods

### 2.1. Plant Material

Kale plants were cultivated according to local agronomic practices in La Plata, Argentina. Prior to crop establishment, the soil was amended with compost produced at the same experimental station. No additional nitrogen fertilization was applied during the crop cycle. A drip irrigation system and polyethylene mulch were installed before transplanting. Plants were arranged in single rows, with a spacing of 50 cm between plants. Irrigation was supplied through the drip system according to crop requirements. Leaves were categorized into three groups: (i) inner leaves (approx. 20 cm in length), representing the most inner, immature developmental stage located at the apex; (ii) middle leaves (20–30 cm), representing an intermediate developmental stage; and (iii) outer leaves (35–40 cm), representing the fully expanded, outer stage located at the basal region of the plant. The chronological age of the leaves at harvest was estimated to be approximately 20 days for inner leaves, 30 days for middle leaves, and 45 days for outer leaves. Sixty leaves from each of the three developmental stages were harvested—only one leaf from each developmental per plant. After harvest, leaves were stored at 20 °C in darkness for six days at 90–95% relative humidity (RH). Samples were collected at 0, 2, 4, and 6 days of postharvest storage. At each sampling time, 15 leaves from each developmental stage were analyzed, corresponding to three biological replicates of five leaves each. After recording fresh weight and determining surface color, the leaves were frozen in liquid nitrogen, powdered, and stored for subsequent analyses.

### 2.2. Weight Loss

Kale leaves were weighed at the initial (day of harvest, D0) and during postharvest storage. Weight loss (%) was calculated as a percentage of the initial value (D0).

### 2.3. Superficial Color

Superficial color was determined using a Minolta Chroma Meter CR-300 colorimeter (Osaka, Japan). Four measurements were performed for each leaf, taking two measurements from each side of the leaf midrib. HUE angle was calculated as h° = tan^−1^ (b/a), when a > 0 and b > 0, or as h° = 180° − tan^−1^ (b/a) when a < 0 and b > 0.

### 2.4. Chlorophyll Content

Approximately 20 mg of powdered tissue was homogenized in 2 mL of dimethylformamide. Samples were then incubated overnight in the dark at room temperature. Tubes were centrifuged at 12,000× *g* for 20 min, and the supernatants were measured at 664 and 647 nm. Total chlorophyll content was calculated according to [14].

### 2.5. Ethanolic Extracts Preparation

A total of 200 mg of powdered tissue was homogenized in 1 mL of 100% ethanol. Tubes were incubated for 1 h at room temperature and then centrifuged at 16,000× *g* for 20 min. The supernatants were recovered and were used to measure phenolic content, flavonoid content, antioxidant capacity, and total and reducing sugar contents.

### 2.6. Phenolic Content

Phenolic content was determined using the Folin–Ciocalteu method as described by [15] with minor modifications. Briefly, 50 μL of the ethanolic extract was mixed with 450 μL of distilled water and 100 μL of Folin–Ciocalteu reagent. After 3 min, 500 μL of 10% *w*/*v* Na_2_CO_3_ was added, and the reaction mixture was incubated in the dark for 1 h. The samples were then centrifuged at 12,000× *g* for 10 min. Absorbance of the supernatants was measured at 760 nm.

### 2.7. Flavonoid Content

Flavonoid content was determined using an adaptation described by [16]. Briefly, the reaction mixture consisted of 100 μL of the ethanolic extract, 610 μL of distilled water, and 30 μL of 5% (*w*/*v*) NaNO_2_. After 5 min, 60 μL of 10% (*w*/*v*) AlCl_3_ was added, and the solution was incubated for 5 min. Next, 200 μL of 1 M NaOH was added; the samples were mixed, and their absorbance was measured at 515 nm.

### 2.8. Antioxidant Capacity Assay

Antioxidant activity was determined using the ABTS radical cation decolorization assay according to [17] with minor modifications. The ABTS•^+^ working solution was prepared by reacting 7 mM ABTS with 2.45 mM potassium persulfate and incubating the mixture for 12–16 h at room temperature in the dark. The solution was diluted with 5 mM phosphate buffer (pH 7.4) to obtain an absorbance of 0.70 ± 0.05 at 734 nm. The assay was performed in 96-well microplates by mixing 25 μL of the ethanolic extract with 250 μL of the diluted ABTS•^+^ solution. Absorbance was measured at 734 nm.

### 2.9. Total and Reducing Sugar Content

Total soluble sugars were determined using the anthrone method according to [15] with minor modifications. Briefly, 100 μL of the ethanolic extract was mixed with 1 mL of anthrone reagent. The reaction mixture was heated at 100 °C for 12 min and then cooled in the dark for 20 min. Absorbance was measured at 625 nm using a CLARIOstar microplate reader (BMG LABTECH, Ortenberg, Germany).

Reducing sugars were quantified using the Somogyi–Nelson method [18]. Briefly, 250 μL of the ethanolic extract was mixed with 250 μL of copper reagent. The reaction mixture was boiled for 10 min and then allowed to cool to room temperature. Subsequently, 125 μL of arsenomolybdate reagent and 625 μL of distilled water were added. After incubation for 30 min in the dark at room temperature, absorbance was measured at 520 nm.

### 2.10. Total and Soluble Protein Content

To determine total protein content, 300 mg of powdered tissue was homogenized in 10 mL of a solution containing 0.1 M NaOH and 10 g L^−1^ sodium dodecyl sulfate (SDS). The mixture was heated at 100 °C for 10 min and then centrifuged at 10,000× *g* for 40 min at 4 °C. The supernatants were collected, and proteins were precipitated by adding five volumes of acetone, followed by incubation at −20 °C for 12 h. The samples were then centrifuged at 13,000× *g* for 10 min at 4 °C. The resulting pellets were resuspended in 200 μL of 0.1 M NaOH, and total protein content was determined using the Lowry method [19] with bovine serum albumin (BSA) as the standard.

To determine soluble protein content, 500 mg of powdered tissue was homogenized in 5 mL of extraction buffer containing 50 mM Tris-HCl, 0.4 mL L^−1^ β-mercaptoethanol, and 2 mM ethylenediaminetetraacetic acid (EDTA; pH 7.5). The homogenate was centrifuged at 10,000× *g* for 10 min at 4 °C, and soluble protein content was determined in the supernatant using the Bradford method [20]. Bovine serum albumin (BSA) was used as the standard.

### 2.11. Chlorophyll Catabolic Gene Expression

Total RNA of kale leaf samples was extracted using TRIzol™ reagent following the manufacturer’s recommended protocol. Purification of RNA and cDNA synthesis were performed by following the procedures described previously [21]. The resulting cDNA was employed as a template for the two-step qPCR reaction using a Step One Plus-Real Time PCR System (Applied Biosystems, Waltham, MA, USA) and FastStart Universal SYBR Green Master (Roche, Basel, Switzerland). Three negative controls were included. The following program was used: one cycle at 95 °C for 10 min, then 40 cycles of 95 °C for 15 s and 60 °C for 1 min. Actin gen (*ACT*) was used as a normalizer, and the primer sequences and accession numbers of *NYC*, *NOL*, *SGR*, *PPH*, *PaO* and *ACT* genes are shown in Supplementary Table S1. Three biological and technical replicates were performed, and three negative controls with Milli-Q water instead of cDNA were included. Relative gene expression was calculated using the 2^−ΔΔCt^ method.

### 2.12. Statistical Analysis

All results were expressed in basis to fresh weight (FW). Data are presented as means ± standard deviation (SD). To compare kale leaves, a two-way analysis of variance (ANOVA) including stages, storage day, and their interaction was performed, followed by Tukey’s honestly significant difference (HSD) post hoc test for multiple comparisons. Statistical analyses were performed using InfoStat software (version 2020; InfoStat Group, National University of Cordoba, Argentina) [22]. Different letters indicate statistically significant differences among means at the 5% significance level (*p* < 0.05).

## 3. Results

Leaf development stage, storage day, or their interaction (leaf stage × storage day) affected weight loss, superficial color, and total chlorophyll content (Table 1).

### 3.1. Weight Loss

Inner leaves exhibited the highest percentage of weight loss after two days of storage (5.36%), significantly exceeding that observed in middle leaves (4.69%) (Table 2). A similar pattern was observed after four days of storage, with inner leaves showing significantly greater weight loss than middle leaves, whereas outer leaves displayed no differences between inner and middle weight loss values. However, at day 6, no significant differences were observed among leaves at different stages.

### 3.2. Superficial Color

**Figure 1:**
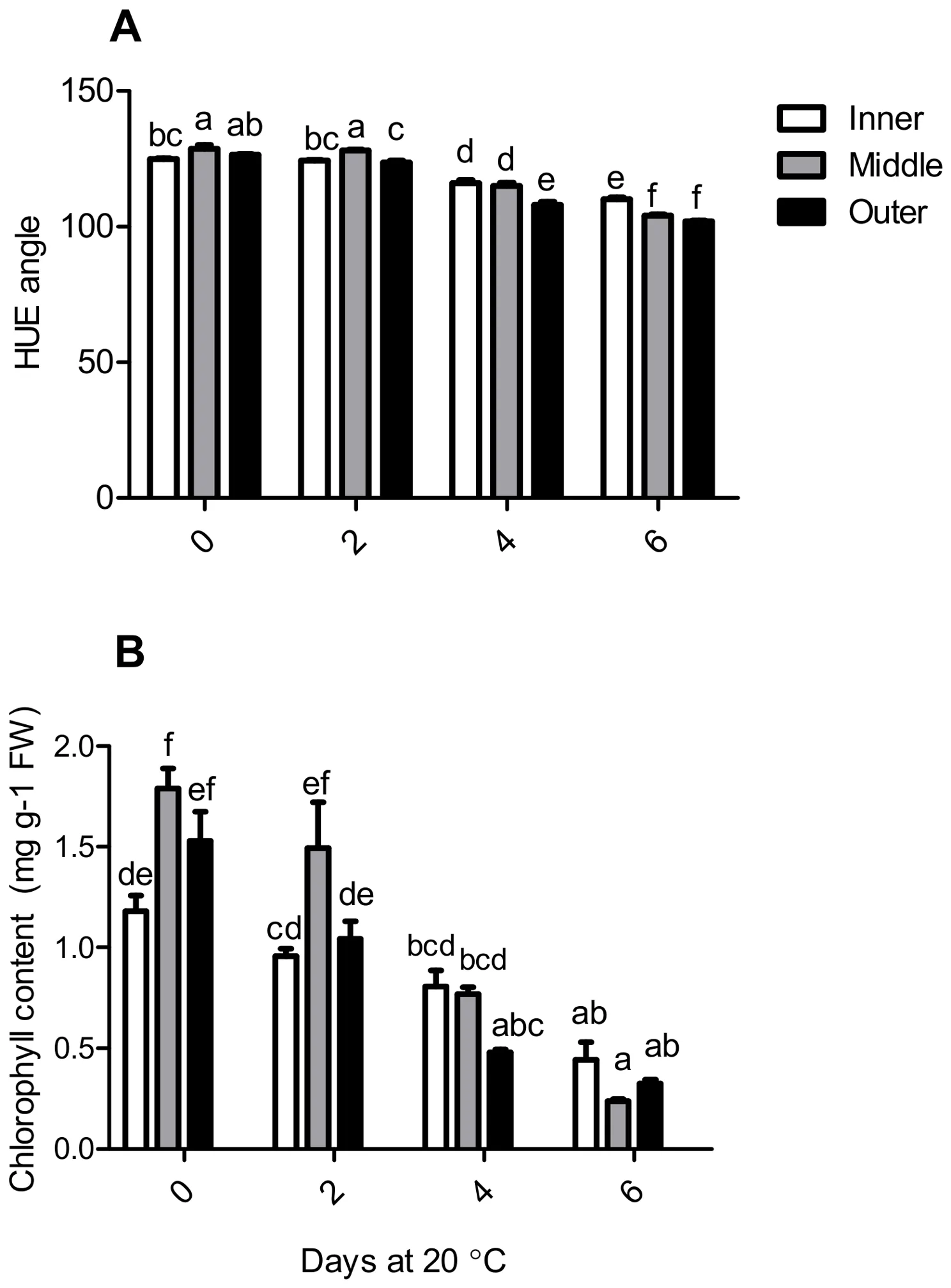
(**A**) Superficial color (HUE angle) and (**B**) content of total chlorophyll expressed as mg of total chlorophyll per gram of fresh weight at day 0, 2, 4 and 6 of inner (white), middle (gray) and outer (black) kale leaves. Data are presented as mean ± standard deviation (SD). Different letters indicate significant differences at different storage times and developmental stages (*p* < 0.05).

The differences in color retention are visually evident in the representative photographs of the leaves at different developmental stages and storage times. During postharvest storage, kale leaves progressively lost their green color and developed a more yellowish appearance, with these changes being more pronounced in middle and outer leaves than in inner leaves. At day 4, inner and middle leaves appear more green than outer leaves. By the end of storage (D6), inner leaves retained a greener appearance compared with middle and outer leaves (Supplementary Figure S2). These visual observations were consistent with the HUE angle measurements. A significant decrease in HUE angle was observed in all leaves during storage, indicating a loss of green color and a transition toward yellowing, a characteristic symptom of leaf senescence. A significant decrease was first observed at day 4 in inner and middle leaves, whereas, in outer leaves, the decrease was already significant at day 2. Consequently, at D4, inner and middle leaves had higher HUE values than the outer leaves, and at D6 inner leaves exhibited the highest HUE angle value (Figure 1A).

### 3.3. Chlorophyll Content and Chlorophyll Degradation Rate

At harvest, the highest total chlorophyll content was observed in leaves at the middle developmental stage (1.8 mg/g FW), whereas inner leaves exhibited the lowest values (1.1 mg/g FW) (Figure 1B). Nevertheless, leaves harvested at the inner developmental stage exhibited the lowest rate of chlorophyll degradation after 4 and 6 days of storage, in comparison with leaves harvested at the middle and outer stages. At day 4 of postharvest storage, the percentage of chlorophyll degradation in outer leaves was nearly twice that observed in inner leaves (Table 3).

Leaf development stage, storage day, or their interaction (leaf stage × storage day) affected antioxidant capacity, phenolic content, total and reducing sugars and total and soluble protein content (Table 4).

### 3.4. Phenolic Content and Antioxidant Capacity

**Figure 2:**
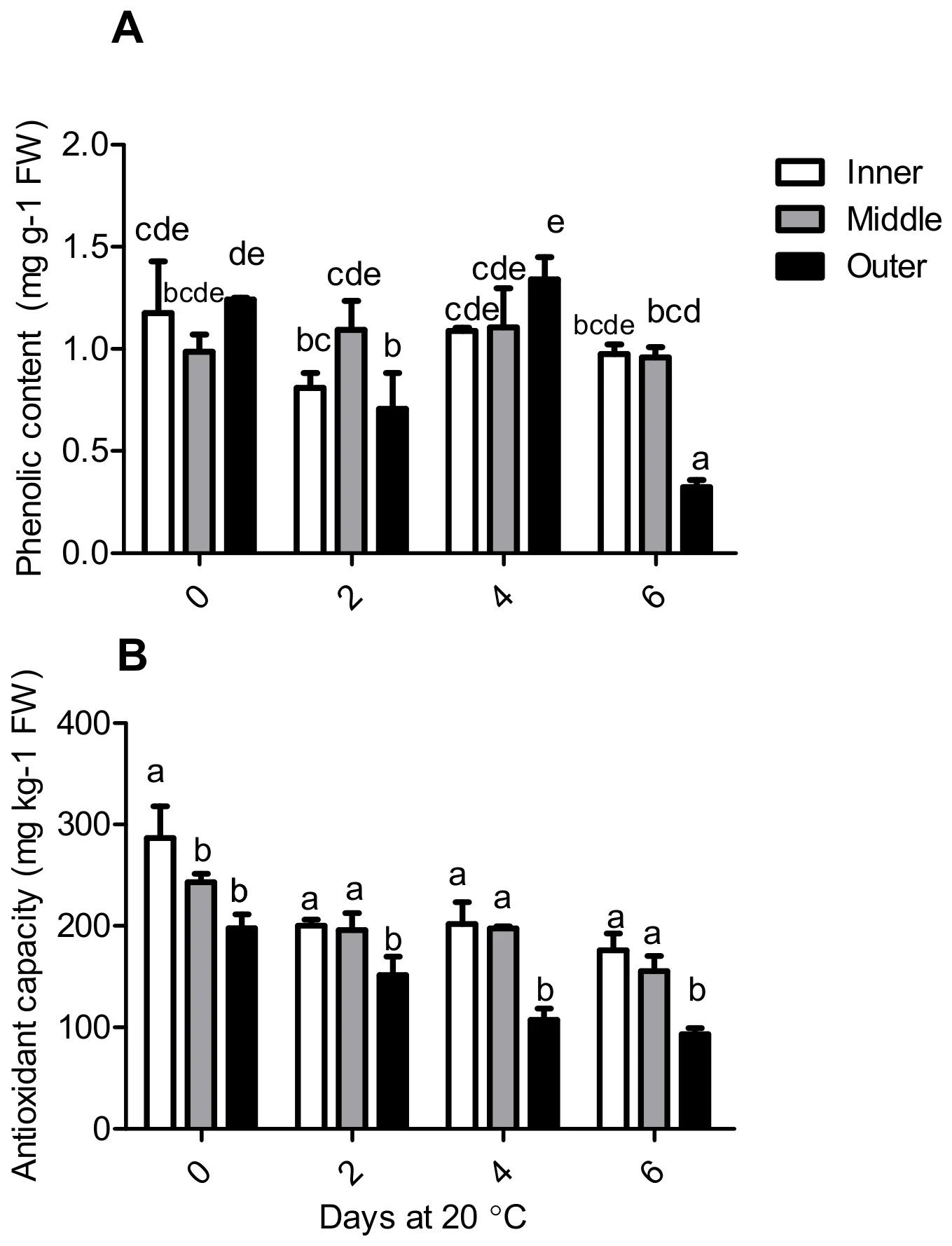
(**A**) Total phenolic content expressed as mg of phenolic content per g of fresh weight (FW). Data are presented as mean ± standard deviation (SD). Different letters indicate significant differences at different storage times and developmental stages (*p* < 0.05). (**B**) Antioxidant capacity expressed as mg per kilogram of fresh weight (FW) at day 0, 2, 4 and 6 for inner (white), middle (gray) and outer (black) kale leaves. Data are presented as mean ± standard deviation (SD). Different letters indicate significant differences at the same time of storage (*p* < 0.05).

With respect to the total phenolic content, at harvest, no differences were found among leaves at different stages, but at the end of storage inner and middle leaves had a higher concentration of phenols than outer leaves (Figure 2A). The flavonoid content did not differ significantly among developmental stages at any of the evaluated storage times (Supplementary Figure S3). Finally, as shown in Figure 2B, at harvest, inner leaves had a higher antioxidant capacity than the other leaves. At day 2, day 4 and day 6, outer leaves exhibited lower values than inner and middle samples.

### 3.5. Sugar Content

**Figure 3:**
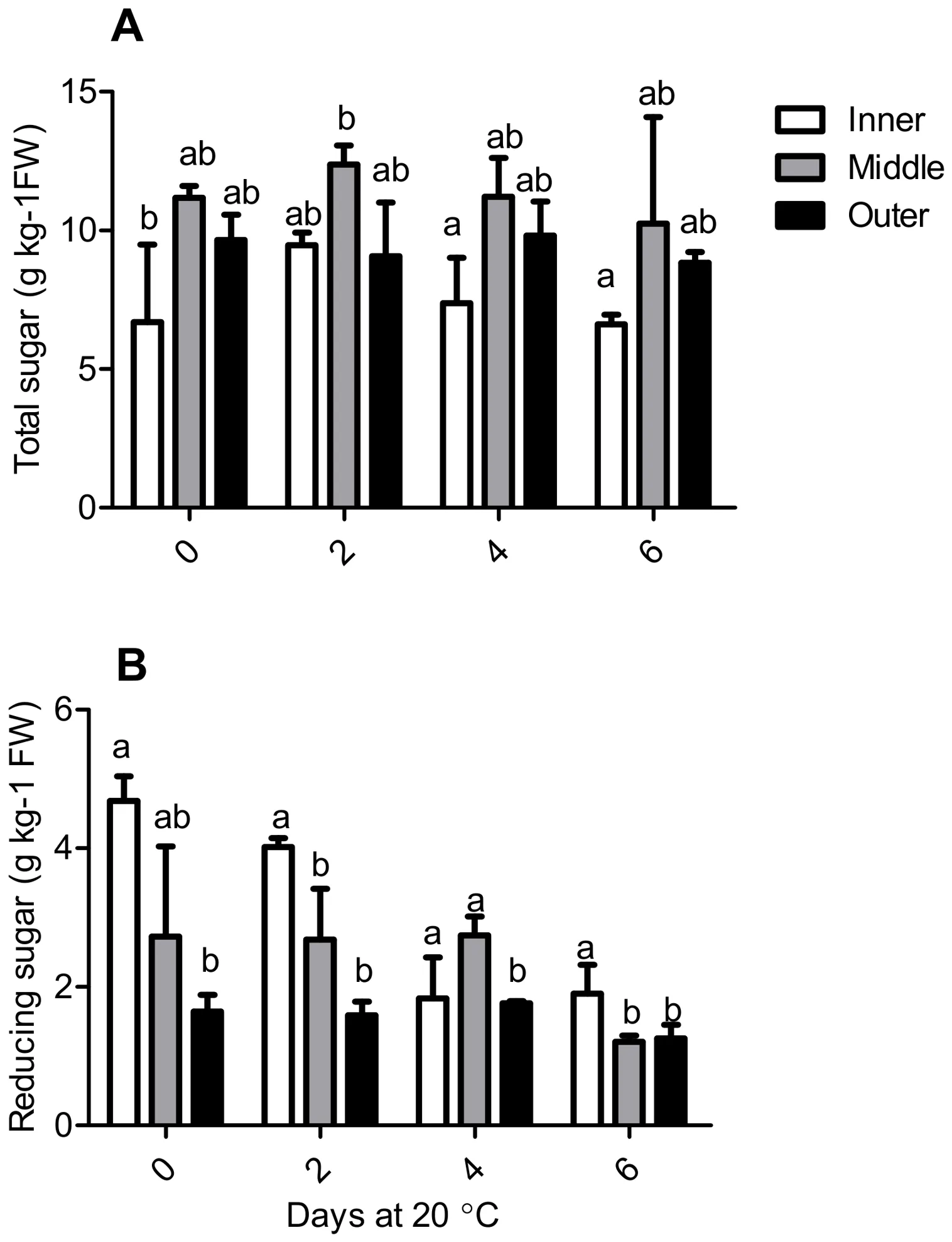
(**A**) Total sugar expressed as g per kilogram of fresh weight (FW) at day 0, 2, 4 and 6 for inner (white), middle (gray) and outer (black) kale leaves. Different letters indicate significant differences at different storage times and developmental stages (*p* < 0.05). (**B**) Reducing sugar expressed as g per kilogram of fresh weight (FW) at day 0, 2, 4 and 6 for inner (white), middle (gray) and outer (black) kale leaves. Data are presented as mean ± standard deviation (SD). Different letters indicate significant differences at the same time of storage (*p* < 0.05).

At harvest and at the end of storage, no significant differences in total sugar content were found among leaves at different developmental stages. In general, no marked differences or consistent trends in total sugar content were observed among developmental stages at any of the storage times evaluated (Figure 3A). However, differences were observed in reducing sugar content (Figure 3B). At the time of harvest, the inner leaves showed a higher concentration of reducing sugars than the other two stages.

### 3.6. Protein Content

**Figure 4:**
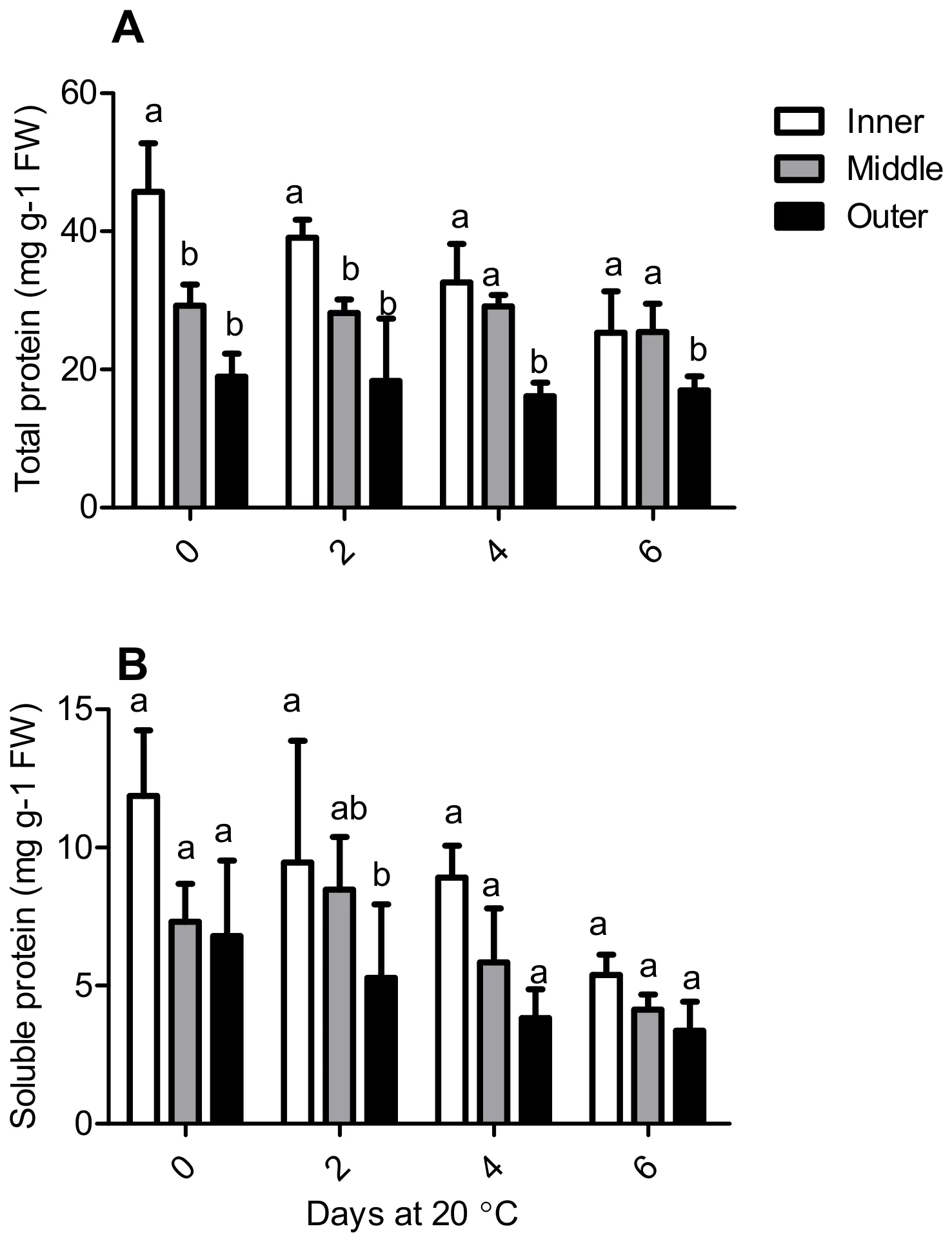
(**A**) Total protein and (**B**) soluble protein expressed as mg of protein per gram of fresh weight (FW) at day 0, 2, 4 and 6 for inner (white), middle (gray) and outer (black) kale leaves. Data are presented as mean ± standard deviation (SD). Different letters indicate significant differences at the same time of storage (*p* < 0.05).

Inner leaves exhibited higher protein concentrations than middle leaves at day 0 and 2, and higher concentrations than outer leaves at harvest and throughout the postharvest storage period (Figure 4A). At harvest, inner leaves contained an average of 45.7 mg protein g^−1^ fresh weight (FW), whereas outer leaves contained approximately 19 mg protein g^−1^ FW. These differences were maintained throughout postharvest storage, with inner leaves still exhibiting approximately twice the total protein content of outer leaves at the end of storage. Regarding soluble proteins, a continuous decline in content was detected in the samples of the three types of leaves during storage, consistent with the protein degradation that occurs during senescence. However, no differences among developmental stages were observed. The only exception occurred on day 2, where outer leaves had a minor concentration of soluble protein (Figure 4B).

### 3.7. Chlorophyll Catabolic Gene Expression

Considering that leaf degreening and yellowing is one of the most important features of leaf senescence, the expression of genes involved in chlorophyll catabolism (CCGs) was analyzed.

Leaf development stage, storage day, or their interaction (leaf stage × storage day) affected *NYC*, *NOL*, *SGR*, *PPH* and *PaO* (Table 5).

**Figure 5:**
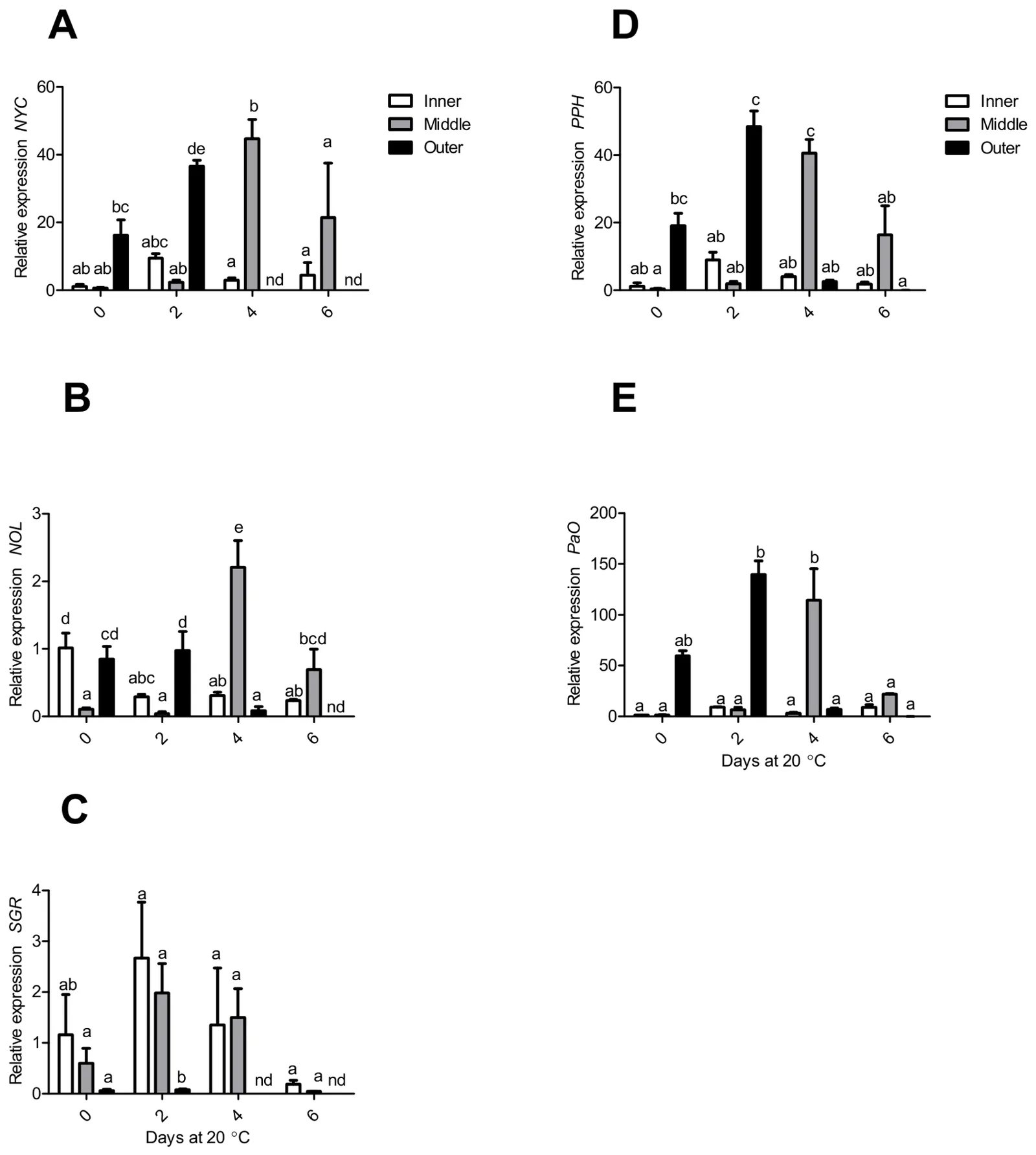
Relative gene expression of (**A**) *NYC*, (**B**) *NOL*, (**C**) *SGR*, (**D**) *PPH* and (**E**) *PaO* at day 0, 2, 4 and 6 for inner (white), middle (gray) and outer (black) kale leaves. nd: no detected. Data are presented as mean ± standard deviation (SD). Different letters indicate significant differences at different storage times and developmental stages (*p* < 0.05).

For *NYC*, *PPH*, and *PaO*, outer leaves exhibited the highest expression levels at harvest (D0) and on day 2 (D2), followed by a sharp decline to very low or undetectable levels by days 4 (D4) and 6 (D6). *NYC* relative expression was approximately 40-fold higher at D2 in outer leaves than in inner and middle leaves. Similarly, *PPH* and *PaO* expressions increased by approximately 48-fold and 140-fold, respectively, by day 2 of storage. Middle leaves exhibited increases of approximately 43-fold for *NYC*, 41-fold for *PPH*, and 110-fold for *PaO*; however, these changes occurred later, on day 4 of storage, followed by a significant decline in expression by day 6 (Figure 5A,D,E). Finally, in inner leaves, the expression levels of *NYC*, *PPH* and *PaO* were very low at D0 and did not present significant changes for the rest of the storage time. Overall, the expression of these genes was highest at D0 and D2 in outer leaves and at D4 and D6 in middle leaves, whereas no increases in its expression were observed in inner leaves.

In the case of *NOL* (Figure 5B), the expression pattern in middle and outer leaves was similar to that described above. However, in inner leaves, a decline in expression was observed throughout D0 and D2. Finally, *SGR* expression was initially higher with respect to the mature stage at day 2 and day 4. In contrast, expression in the outer leaves remained very low or undetectable throughout the storage period (Figure 5C).

## 4. Discussion

The leaf developmental stage at harvest strongly influenced the postharvest behavior of kale leaves, particularly the progression of senescence and the maintenance of visual and nutritional quality parameters and pigments [23,24]. A previous study similarly reported greater deterioration and discoloration in outer kale leaves during refrigerated postharvest storage compared with leaves harvested at middle and inner developmental stages [13]. Differences in the physiological status of leaves at harvest may contribute to these distinct postharvest responses [25,26]. Among the hormones involved in the regulation of senescence, cytokinins play an important role as negative regulators of the process. Previous studies have shown that endogenous cytokinin concentrations are higher in inner, expanding leaves, where they support cell division and sink strength, whereas cytokinin levels progressively decline in outer and basal leaves undergoing senescence [27,28]. This developmental gradient in hormonal status may contribute to the different capacity of leaves to delay senescence after harvest. The inner developmental stage is characterized by immature, metabolically active tissues in which photosynthetic structures, such as chloroplasts, are still developing. This may explain the lower total chlorophyll content observed in inner leaves at harvest. Despite this, inner leaves maintained better visual quality during storage and showed a lower degree of chlorophyll degradation, whereas outer leaves exhibited a more pronounced loss of green color and a greater progression of senescence. Thus, the developmental status of the leaf at harvest appears to be related to its subsequent postharvest behavior. Differences among developmental stages were also observed for weight loss, particularly during the early stages of storage. Inner leaves exhibited greater weight loss than middle and outer leaves during the first days of storage. This response may be related to differences in leaf morphology and physiological characteristics that influence water loss through transpiration. The cuticle of inner leaves is less developed, which may reduce its effectiveness as a barrier against non-stomatal water loss [29]. In agreement with this interpretation, previous studies have shown that inner leaves exhibit a higher minimum cuticular conductance and greater surface wettability than outer leaves, characteristics that may facilitate foliar water absorption but also increase water loss [30,31]. Differences in metabolic activity may also contribute to the initial differences in weight loss, as inner leaves have been reported to exhibit higher respiration rates than more fully developed leaves [13]. Similar differences in respiratory activity have been reported in rocket leaves, in which inner leaves exhibited higher respiration rates than more outer leaves [11]. As storage progressed, however, differences in weight loss among developmental stages were no longer detected. This convergence suggests that the initial differences in water loss and metabolic activity become less pronounced as postharvest senescence progresses. The effect of developmental stage on nutritional characteristics was less uniform than that observed for visual quality and chlorophyll degradation. The concentration and profile of phenolic compounds in vegetables depend on both genetic factors and environmental conditions, including biotic factors, such as herbivory, and abiotic factors, such as light intensity and quality, temperature, and nutrient availability, among others [32]. Phenolic compounds and antioxidant capacity can vary during leaf development, although the direction and magnitude of these changes are highly dependent on species, genotype, and environmental conditions [33,34]. In the present study, flavonoid content did not differ significantly among developmental stages throughout storage, suggesting that this group of compounds was relatively stable with respect to leaf developmental stage under the conditions evaluated.

Sugar content is closely associated with leaf senescence and may play an important role in the regulation of senescence processes in green tissues [35]. In contrast, reducing sugars showed a clear developmental pattern. Inner leaves had higher reducing sugar concentrations at harvest but experienced a marked decline during storage, whereas the decrease was less pronounced in middle leaves and was not detected in outer leaves. The higher metabolic activity generally associated with developing tissues may contribute to this pattern. Inner leaves are metabolically active tissues with high respiratory demand, and the greater utilization of reducing sugars to support respiration and other energy-demanding processes associated with growth and tissue maintenance may explain their pronounced decline during storage [11,24,25]. Conversely, the relatively stable reducing sugar content of outer leaves may reflect their lower metabolic demand at this developmental stage. These results suggest that developmental stage affects not only the initial concentration of specific metabolites but also their utilization during postharvest storage. Protein content provided further evidence of differences in the physiological status of the leaves at harvest and their subsequent response to storage. Protein degradation is a characteristic feature of leaf senescence and is associated with the mobilization and remobilization of nitrogen and other nutrients from senescing tissues [36]. Similar reductions in protein content have been reported during postharvest senescence in broccoli [37] and have been associated with increased protease activity in *Brassica* species [38]. In the present study, protein content decreased during storage, particularly in inner leaves, which had the highest protein concentration at harvest. In contrast, middle and outer leaves, which had lower protein concentrations at harvest, showed comparatively minor changes throughout storage. This pattern suggests that the developmental stage at harvest influences not only the initial protein status of the tissue but also the extent to which protein reserves are mobilized during postharvest storage. Nitrogen availability during crop development may also contribute to differences in leaf size, chlorophyll accumulation, and green color among developmental stages. Nitrogen is an essential component of chlorophyll and is closely associated with photosynthetic capacity and vegetative growth. Therefore, differences in nitrogen status during leaf development could contribute to the differences in size and chlorophyll content observed among the leaves at harvest. In the present study, middle leaves had the highest chlorophyll content at harvest, whereas inner leaves, despite their smaller size and lower initial chlorophyll content, subsequently showed greater chlorophyll retention and greener color during storage. The higher protein content observed in inner leaves may also be consistent with differences in nitrogen allocation among tissues at different developmental stages.

Taken together, the results obtained for visual quality, chlorophyll degradation, sugars, and proteins suggest that developmental stages differ not only in the magnitude of the senescence response but also in the timing of its progression. This distinction is particularly evident from the expression patterns of genes involved in chlorophyll catabolism. The transcriptional activation of *NYC*, *PPH*, and *PaO* has been reported during senescence in *Brassica* species and *Arabidopsis*, supporting their conserved role in the chlorophyll catabolic pathway [9,39]. In the present study, these genes exhibited markedly different temporal expression patterns depending on leaf developmental stage. Outer leaves showed relatively high transcript levels of *NYC*, *PPH*, and *PaO* at harvest or during the first two days of storage, whereas the induction of these genes in middle leaves occurred mainly at later stages of storage. In contrast, inner leaves did not show a comparable increase within the sampling period. These results suggest that the molecular program associated with chlorophyll degradation is activated earlier in outer leaves, becomes activated later in middle leaves, and is delayed or less pronounced in inner leaves. The expression pattern of *NYC* provides particularly strong evidence for this temporal shift. Although *NYC* expression was markedly higher in outer leaves at harvest, middle leaves showed a pronounced increase during the later stages of storage, whereas inner leaves did not exhibit a comparable induction. This pattern may indicate that senescence-associated chlorophyll catabolism was already activated in outer leaves at or before harvest, while its transcriptional activation was progressively delayed in the younger developmental stages. A similar temporal shift was observed for *PPH* and *PaO*, which showed earlier induction in outer leaves and later increases in middle leaves. The expression of *SGR* followed a related developmental pattern, although its transcript accumulation was not detected as an increase in outer leaves during the storage period. One possible explanation is that *SGR* induction occurred before harvest and had already declined by the time of sampling. However, this possibility cannot be confirmed without measurements at additional preharvest developmental stages. Importantly, the absence of a clear induction of these genes in inner leaves, together with their lower chlorophyll degradation and better maintenance of green color, is consistent with a delayed progression of the senescence-associated chlorophyll catabolic program. The expression pattern of *NOL*, which encodes the other chlorophyll b reductase involved in the conversion of chlorophyll b to chlorophyll a, was less pronounced than that of *NYC*. This observation is consistent with previous reports indicating that *NOL* does not generally show a strong increase in expression during induced senescence [40], suggesting that *NYC* may have a more prominent role than *NOL* in chlorophyll catabolism during senescence [41]. The correspondence between gene expression and physiological changes further supports the interpretation of a temporally shifted senescence-associated chlorophyll catabolism. Outer leaves, which exhibited an earlier expression of several chlorophyll catabolism genes, also showed greater chlorophyll degradation and a more pronounced loss of green color during storage. Middle leaves showed a later increase in the expression of several of these genes, coinciding with the progression of chlorophyll degradation at later stages of storage. In contrast, inner leaves showed limited transcriptional activation of these genes during the experimental period and retained greater green color and chlorophyll content. Thus, the differences among developmental stages appear to reflect a temporal shift in the activation of senescence rather than simply different rates of the same response. This delayed activation of the chlorophyll catabolic program in inner leaves may contribute to their greater capacity to maintain visual quality during postharvest storage. Finally, the decline in the expression of several chlorophyll catabolism genes at D4 and D6, particularly in outer and middle leaves, should be interpreted with caution. A reduction in transcript abundance does not necessarily indicate that chlorophyll degradation has ceased or that the downstream catabolic pathway is no longer active. Advanced senescence is accompanied by progressive cellular deterioration and may also involve changes in RNA stability and overall transcriptional activity. Moreover, because enzymatic activities and protein abundance were not directly quantified in the present study, pre-existing catabolic enzymes may remain stable and functionally active despite the decline in transcript levels. Therefore, the later decrease in transcript abundance may reflect a transition from active transcriptional induction toward more advanced stages of senescence rather than a termination of chlorophyll catabolism. The differences observed among leaves of developmental stages indicate that the postharvest behavior is closely associated with their physiological status at harvest. The delayed activation of senescence-associated chlorophyll catabolism in inner leaves is consistent with a less advanced senescence state, whereas the earlier molecular response observed in outer leaves suggests that these tissues are closer to the onset of senescence at the time of harvest. The combined physiological and transcriptional patterns support the interpretation that developmental status at harvest influences the timing of postharvest senescence in kale leaves.

## 5. Conclusions

Overall, the results demonstrate that the developmental stage at harvest influences the postharvest quality and senescence progression of kale leaves stored at room temperature. The differences among inner, middle, and outer leaves reflect the combined effects of canopy position, leaf size, and physiological maturity rather than chronological age alone. Inner leaves showed greater postharvest stability, with better retention of green color, lower chlorophyll degradation, and higher protein levels, together with a delayed activation of genes related to chlorophyll catabolism. These findings indicate that harvest stage is a practical factor that can be considered to improve kale postharvest quality. Selecting leaves at earlier developmental stages may represent a simple, low-cost strategy to delay senescence and maintain nutritional quality during postharvest storage.

## Figures and Tables

**Table 1:** Effects of leaf developmental stage, storage day, and their interaction on weight loss, superficial color, and chlorophyll content.

Source	Weight Loss	Superficial Color	Chlorophyll Content
Leaf stage	*	*	*
Storage day	*	*	*
Leaf stage × storage day	NS	*	*

Note: NS, not significant; * indicates significant differences according to analysis of variance (ANOVA) at *p* ≤ 0.05.

**Table 2:** Percentage of weight loss at day 2, day 4 and day 6 expressed as percentage with respect to day 0. Data are presented as mean ± standard deviation (SD). Different letters indicate significant differences (*p* < 0.05) among samples at the same time.

Leaf Stage	Day 2	Day 4	Day 6
Inner	5.36 ± 0.30 b	9.99 ± 0.21 b	16.1 ± 0.21 a
Middle	4.69 ± 0.19 a	8.91 ± 0.40 a	15.5 ± 0.65 a
Outer	4.85 ± 0.19 a	9.52 ± 0.22 ab	16.6 ± 0.60 a

**Table 3:** Chlorophyll degradation rate after 2, 4 and 6 days of postharvest storage at 20 °C, expressed as percentage with respect to day 0. Data are presented as mean ± standard deviation (SD). Different letters indicate significant differences (*p* < 0.05) among samples at the same time.

Leaf Stage	Day 2	Day 4	Day 6
Inner	18.87 ± 5.17 a	31.75 ± 11.69 a	62.56 ± 13.08 a
Middle	26.53 ± 18.74 a	56.98 ± 3.22 b	86.72 ± 0.96 b
Outer	37.36 ± 0.77 a	68.61 ± 1.56 b	78.83 ± 2.25 b

**Table 4:** Effects of leaf developmental stage, storage day, and their interaction on antioxidant capacity, phenolic content, total and reducing sugars, and total and soluble protein.

Source	Antioxidant Capacity	Phenolic Content	Total Soluble Sugars	Reducing Sugars	Total Protein	Soluble Protein
Leaf stage	*	*	*	*	*	*
Storage day	*	*	*	*	*	*
Leaf stage × Storage day	NS	*	*	NS	NS	NS

Note: NS, not significant; * indicates significant differences according to analysis of variance (ANOVA) at *p* ≤ 0.05.

**Table 5:** Effects of leaf developmental stage, storage day, and their interaction on gene expression of *NYC*, *NOL*, *SGR*, *PPH* and *PaO*.

Source	*NYC*	*NOL*	*SGR*	*PPH*	*PaO*
Leaf stage	*	*	*	*	*
Storage day	*	*	*	*	NS
Leaf stage × Storage day	*	*	*	*	*

Note: NS, not significant; * indicates significant differences according to analysis of variance (ANOVA) at *p* ≤ 0.05.

## Data Availability

The original contributions presented in this study are included in the article or in Supplementary Material. Further inquiries can be directed to the corresponding author.

## Supplementary Materials

The following supporting information can be downloaded at: https://www.mdpi.com/article/10.3390/metabo16090684/s1, Figure S1: Chlorophyll catabolic pathway during leaf senescence; Figure S2: Representative photographs of kale leaves at different developmental stages during postharvest storage; Figure S3: Total flavonoid content; Table S1: Primers used for the relative gene expression analysis of chlorophyll catabolism-related genes in kale leaves [42].
