# Supplementary material for: Leaf Developmental Stage at Harvest Affects Postharvest Senescence and Quality Parameters of Kale
Source: Metabolites. 2026 Sep 17;16(9):684. doi: 10.3390/metabo16090684 (PMC13609403; doi:10.3390/metabo16090684)
Supplement: Supplementary file 1 [file metabolites-16-00684-s001.zip › metabolites-4529877-supplementary.pdf]

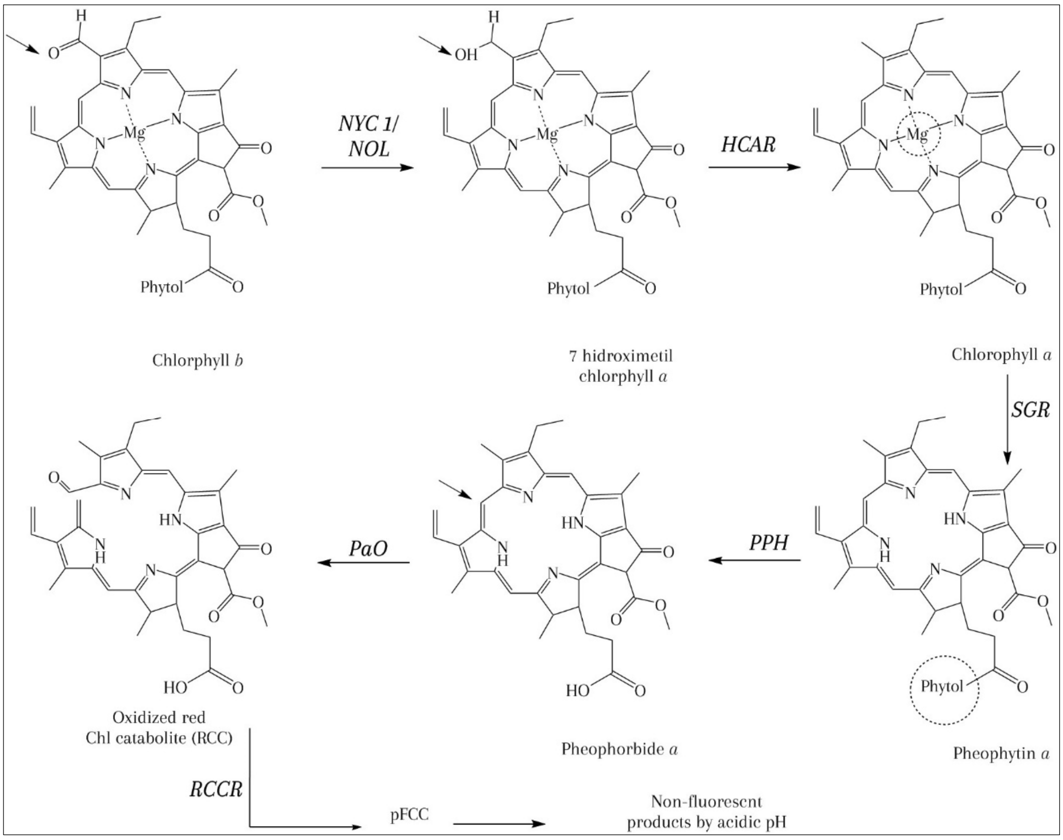

Figure S1. Chlorophyll catabolic pathway during leaf senescence. Schematic representation of chlorophyll degradation, showing the conversion of chlorophyll b to 7-hydroxymethyl chlorophyll a mediated by chlorophyll b reductases encoded by NYC1 and NOL, followed by conversion to chlorophyll a by HCAR. Chlorophyll a is subsequently converted to pheophytin a by SGR, followed by hydrolysis of the phytol chain by PPH to produce pheophorbide a. PaO catalyzes the formation of oxidized red chlorophyll catabolites (RCCs), which are further converted by red chlorophyll catabolite reductase (RCCR) into primary fluorescent chlorophyll catabolites (pFCCs) and subsequently into non-fluorescent chlorophyll catabolites under acidic conditions.

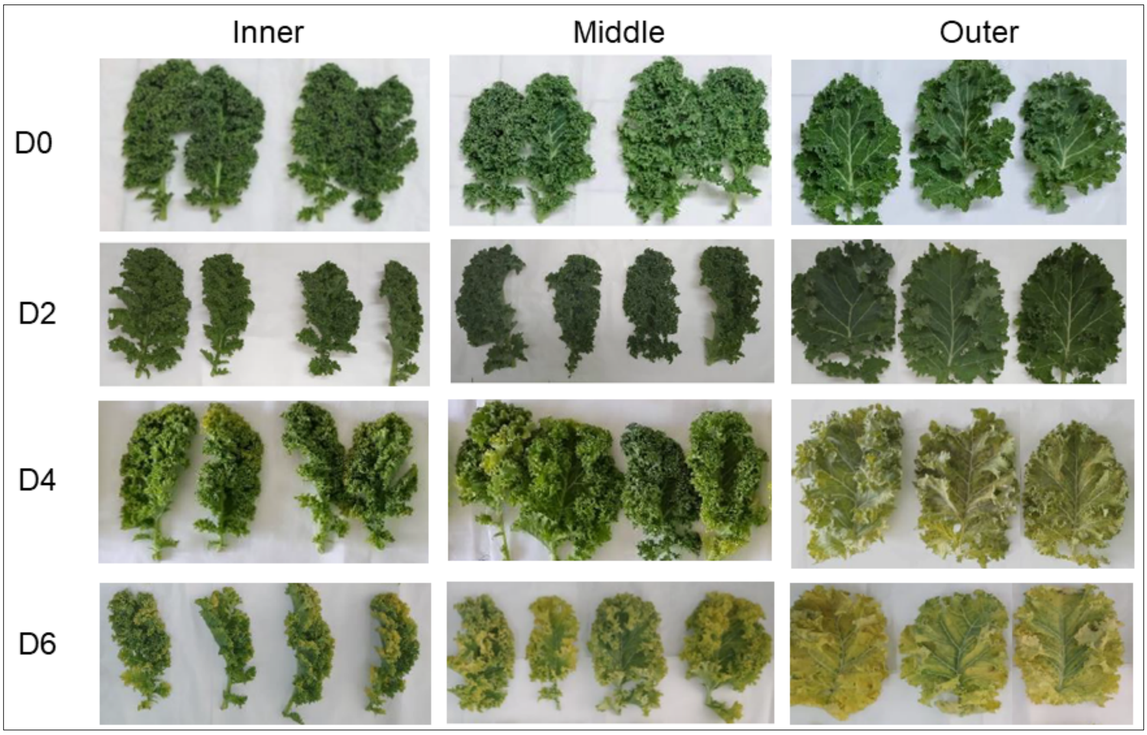

Figure S2. Representative photographs of kale leaves at different developmental stages during postharvest storage. Representative images of inner, middle, and outer leaves at harvest (D0) and after 2 (D2), 4 (D4), and 6 (D6) days of storage at 20 °C in darkness.

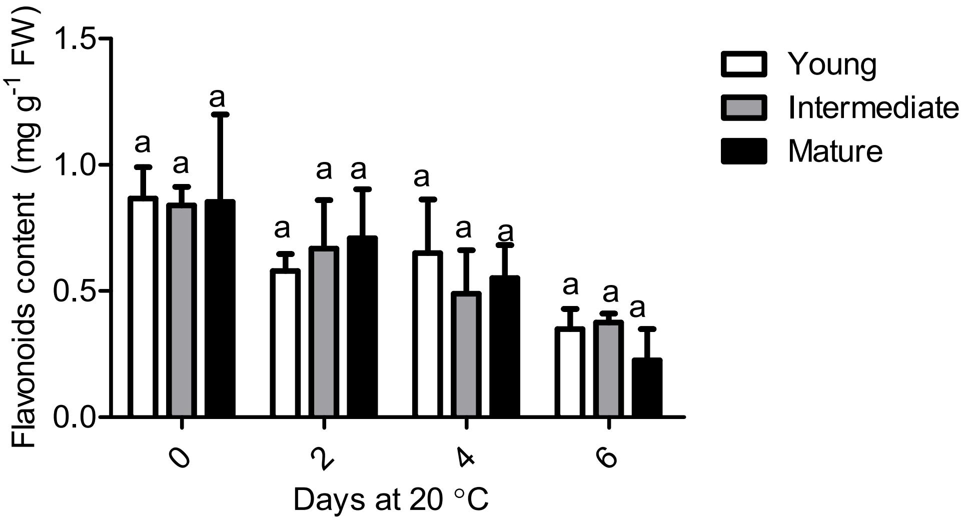

Figure S3. Total flavonoids content expressed as mg per g of fresh weight (FW) at day 0, 2, 4 and 6 of young (White), intermediate (gray) and mature (black) kale leaves. Data are presented as mean ± standard deviation (SD). Different letters indicate significant differences at the same time of storage ($p < 0.05$).

Table S1. Primers used for the relative gene expression analysis of chlorophyll catabolism-related genes in kale leaves. The table includes the forward and reverse primer sequences, amplicon size in basis pairs, and corresponding NCBI accession numbers [35] for *NYC*, *NOL*, *SGR*, *PPH*, and *PaO*, as well as *ACT*, which was used as the reference gene [42].

**Gene**	**Forward primer**	**Reverse primer**	**Amplicon size (bp)**	**NCBI accession number**
NYC	TTACATCTCGCAGTTCTGA	GCAATACCAACTACCTTAGC	130	XM_013780573
NOL	ATGGCGGGGAAGGAGGACAGTGA	GGCGACGGTTGGGGAGGTGATTTA	159	XM_013771448
SGR	TTCCGACAACCGAAGTAA	GTGAGCGTATAAGTTCTTGG	198	XM_013748873
PPH	AGAGGTTATCGGTGAGCCA	GACGGATGAGGATGGG	91	XM_013760640
PaO	CTCTTCTTCCTCTTCCTTC	GACCCTTCTTCTTCCTTATC	315	XM_013752085
ACT	CCAGAGGTCTTGTTCCAGCCATC	GTTCCACCACTGAGCACAATGTTAG	137	XM_01373137

35 Kim J. Sugar metabolism as input signals and fuel for leaf senescence. Genes Genomics. 2019;41(7):737-746.

42 National Center for Biotechnology Information. National Center for Biotechnology Information [Internet]. Bethesda (MD): National Library of Medicine (US); [cited 2024 Oct 23]. Available from: https://www.ncbi.nlm.nih.gov/
